# Alcohol Mixed with Energy Drinks (AmED) and Negative Alcohol-Related Consequences among South Korean College Students

**DOI:** 10.3390/ijerph16071127

**Published:** 2019-03-29

**Authors:** Sarah Soyeon Oh, Yeong Jun Ju, Eun-Cheol Park, Sung-In Jang

**Affiliations:** 1 Institute of Health Services Research, Yonsei University, Seoul 03722, Korea; sarahoh@yuhs.ac (S.S.O.); joomeon@gmail.com (Y.J.J.); ecpark@yuhs.ac (E.C.P.); 2 Department of Public Health, Graduate School, Yonsei University, Seoul 03722, Korea; 3 Department of Preventive Medicine and Public Health, Ajou University, Suwon 16499, Korea; 4 Department of Preventive Medicine, Yonsei University College of Medicine, Seoul 03722, Korea

**Keywords:** energy drinks, alcohol, risk taking, college drinking, AmED, alcohol-related consequences

## Abstract

Consumption of alcohol mixed with energy drinks (AmED) has been associated with various alcohol-related consequences among college students. However, more information is required to assess how this relationship is affected by sociodemographic and environmental factors. This paper investigates the association between AmED consumption and negative alcohol-related consequences while (1) stratifying AmED users by sex, (2) examining a range of outcomes specific to the college context (e.g., missing class), and (3) controlling for drinking frequency and amount. We surveyed and analyzed the data of 4592 students in a nationally representative sample of 82 colleges in South Korea. Multiple linear regression analysis was used to identify the association between AmED use and a number of alcohol-related consequences (ranging from a score of 0–12) while adjusting for covariates, including drinking frequency and intake per drinking session. Of our study population, 22.0% of alcohol-consuming men and 13.4% of alcohol-consuming women reported AmED consumption in the past 12 months. AmED users experienced a greater number of alcohol-related consequences (e.g., missing class, engaging in unplanned sexual activity) than non-AmED users (men β: 0.804, *p* ≤ 0.0001; women β: 0.522, *p* ≤ 0.0001). Male AmED users consuming alcohol once a month (β: 1.155, *p* ≤ 0.0001) and female users consuming less than once a month (β: 1.019, *p* ≤ 0.0001) experienced the greatest number of consequences compared to non-users, as did AmED users consuming 3–4 drinks per drinking session (men β: 1.012, *p* ≤ 0.0001; women β: 0.993, *p* ≤ 0.0001). Our findings reveal that both male and female college students who consume AmED experience a greater number of negative alcohol-related consequences than those who do not. Rather than high-risk drinkers, moderate drinkers who consume alcohol infrequently and/or in low amounts may experience more consequences when consumers of AmED.

## 1. Introduction

Alcohol consumption among college students contributes to a range of negative alcohol-related consequences. In the United States each year, approximately 1825 deaths (e.g., from homicides, motor-vehicle crashes, and suicides), 97,000 sexual assaults, and 600,000 injuries are alcohol-related [1]. In South Korea, around 10.8% of deaths among college students are attributed to alcohol, while more than 50% of colleges experience alcohol-related problems such as campus vandalism and violence by intoxicated students [2].

Recent investigations have highlighted the potential association between alcohol mixed with energy drinks (AmED) and negative alcohol-related consequences among college students. AmED consumption occurs when individuals co-ingest alcohol and energy drinks, i.e., beverages that contain high levels of caffeine, ranging from 50 to 500 mg or more per can [3]. Of college students, 15–34% consume AmED [4] for reasons ranging from “to hide the flavor of alcohol” and/or “to drink more” to “to feel/look less drunk” [5]. AmED consumption has been associated with increased odds of driving a car under the influence, being hurt or injured, experiencing unwanted sexual contact, having unprotected sex, and using drugs [6]. Wolfson and colleagues found that college students who consume AmED are two times more likely to take advantage of another individual sexually (odds ratio (OR): 2.18, 95% CI: 1.34–3.55) and/or require medical treatment (OR: 2.17, 95% CI: 1.24–3.80) than non-consumers [7]. In an online survey of 704 college students, Snipes and colleagues found that monthly AmED consumers are more likely to use marijuana, ecstasy, and cocaine [8]. Peacock and colleagues established that consequences of AmED consumption are always multi-dimensional (physiological: speech and walking difficulties, nausea, slurred speech; psychological: tension, irritability); physiological and psychological symptoms interact with one another to increase odds of impulsive behaviors and accidents [8].

Regarding alcohol consumption frequency and amount, there have been varying results regarding AmED’s influence. Verster and colleagues claim that AmED consumption decreases the frequency and quantity of alcohol consumed, even when drinking occasions and phenotypical differences are controlled for [9]. Animal studies have indicated that certain ingredients contained in energy drinks, such as taurine, can reduce subsequent alcohol intake [10]. More studies, however, have noted that AmED consumption induces hazardous and risky drinking behaviors [≥8 on Alcohol Use Disorders Identification Test (AUDIT)] [4,11], especially with regard to drinking frequency, amount, and duration [4,12,13,14].

Thus, this paper goes beyond previous research by investigating AmED consumption and alcohol-related consequences while (1) stratifying AmED users by sex, (2) examining a range of outcomes specific to the college context, and (3) controlling for drinking frequency and amount. Because consumption of AmED has been gaining popularity among college students in recent years, more information is required to assess how this relationship is affected by sociodemographic and environmental circumstances.

In particular, few studies have examined a range of negative alcohol-related consequences that scholars from the Harvard College Alcohol Study have deemed relevant to the college context, i.e., have a hangover, do something you regret, miss a class, forget where you were or what you did, get behind in school work, argue with friends, engage in unplanned sexual activity, get hurt or injured, damage property, be sexually assaulted, get into trouble with campus or local police, require medical treatment [15]. Therefore, the present study aims to determine the association between AmED consumption and negative alcohol-related consequences among college students while controlling for drinking behavior.

## 2. Materials and Methods

### 2.1. Study Population and Data

In the 2017 national statistics published by the Korean Educational Development Institute on college students, 1,951,940 students (four-year courses of study: 1,506,745; two-year courses of study: 445,195) were enrolled in 356 colleges (four-year courses of study: 195; two-year courses of study: 161) in South Korea [16]. From these colleges, we excluded 23 that had fewer than 500 administered students or were located in the remote island of Jeju. Of the remaining 333 colleges, we randomly selected 85 colleges for our survey analyses. During the recruitment process, three four-year colleges declined to participate in our survey for religious reasons, decreasing the total number of colleges in our investigation to 82 (four-year: 54; two-year: 28). From these colleges, we stratified a proportionately representative sample of undergraduate students belonging to each province in the country: Seoul/Incheon/Gyeonggi, Gangwon, Daejeon/Chungjeong, Gwangju/Jeolla, Daegu/Gyeongbuk, Busan/Ulsan/ Gyeongnam.

Data were collected via face-to-face surveys with interviewers randomly selecting students passing by each campus’s department buildings. Data collectors were instructed to survey around 60 students from each campus—three males and three females from 10 different majors. Teams of collectors were trained for consistency by Gallup and researchers of our investigation. Each question of the questionnaire was required to be administered orally in a face-to-face manner at an enclosed space such as a café or a lecture room.

In total, 5000 students completed our survey instrument. The response rate was 68.7%, with the total number of approached participants being 7278. A financial incentive of 10,000 Korean Won (equivalent to around 9 U.S. dollars) was given to each participant upon completion of the 14-page survey instrument. For the purpose of this investigation, we excluded 211 students who reported never consuming a sip of alcohol in their entire lives, and 197 students who reported not consuming a single drink in the last 12 months.

The survey instrument asked students a number of questions about their drinking behavior, health, and thoughts on campus-alcohol policy. Whenever possible, the instrument included alcohol-related questions that had been previously given in other international, national, or large-scale epidemiological studies, including the Harvard College Alcohol Study, the Korea National Health and Nutrition Examination Survey (KNHANES), and the Korea Youth Risk Behavior Web-Based Survey (KYRBS).

Following the standards of the Korea Centers for Disease Control & Prevention, a standard drink was defined as the amount of alcohol contained in one standard drinking glass of alcohol (approximately 8 grams of pure alcohol), equivalent to: 1 shot of soju, 1 glass of bottled beer, 2/3 of a canned beer, 1/2 glass of draft beer, 1/2 bowl of makgeolli (rice wine), 1/2 glass of wine, 1 glass of whiskey, 1 shot of cheongju (refined rice wine), 1 shot of herbal liquor, 1 shot of fruit wine, or a 3/5 glass of mixed liquor (soju+beer) [17].

All subjects gave their informed consent for inclusion before they participated in the study. The study was conducted in accordance with the Declaration of Helsinki, and the protocol was approved by Yonsei University Health System’s Institutional Review Board (Y-2017-0084).

### 2.2. Measures

#### Outcome Variable

The number of health and behavioral consequences experienced from alcohol consumption was selected as the outcome variable. The health and behavioral consequences measured were given in the original format of the Weschler questionnaire that was part of the Harvard College Alcohol Study [16]. The questionnaire consisted of the following 12 alcohol-related problems: (1) have a hangover, (2) do something you regret, (3) miss a class, (4) forget where you were or what you did, (5) get behind in school work, (6) argue with friends, (7) engage in unplanned sexual activity, (8) get hurt or injured, (9) damage property, (10) be sexually assaulted, (11) get into trouble with campus or local police, (12) require medical treatment. Individuals were asked to answer the following question: “In the past 12 months, have you ever experienced the following problems from consuming alcohol?” If respondents answered, “Yes,” they were coded as ‘1’; if they answered “No,” they were coded as ‘0’. Responses to all 12 problems were summed to give a possible score ranging from 0 (experience of none of the problems above) to 12 (experience of all 12 problems). This questionnaire has been employed by studies that assess the negative consequences of alcohol consumption in college students in association with alcohol consumption patterns [7,17]. Internal consistency, as measured with Cronbach’s alpha, was acceptable for the total score of the scale (Cronbach’s alpha = 0.75).

### 2.3. Alcohol Mixed with Energy Drinks (AmED)

AmED consumption experience was determined via the following question: “In the past 12 months, have you consumed energy drinks (like Hotsix, Red Bull, etc.) while consuming alcohol?” Respondents who answered “Yes” were categorized as AmED consumers, while respondents who answered “No” were categorized as non-AmED consumers.

### 2.4. Statistical Analysis

Descriptive analyses were used to examine the distribution of general characteristics among study subjects. Frequencies and row percentages were calculated for each variable, and χ^2^ tests were performed to identify correlations between variables. To compare the average values and standard deviations for negative alcohol-related consequences, ANOVA was performed. Multiple linear regression analysis was performed to identify the association between AmED consumption experience and number of negative alcohol-related consequences while controlling for the following factors: alcohol consumption frequency, number of drinks per drinking session, year level, major, grade point average (GPA), spending allowance, smoking status, stress level, depressive thoughts, suicidal thoughts, suicidal attempt, underage drinking experience, and number of clubs/organizations. Also, an examination of alcohol consumption frequency and number of drinks per drinking session according to the number of negative alcohol-related consequences stratified by AmED use was conducted for our subgroup analyses while controlling for the same factors.

All analyses were stratified by sex so that men and women were analyzed separately. The calculated *p*-values in this study were considered significant if lower than 0.05. All analyses were performed using SAS software, version 9.4 (SAS Institute, Cary, NC, USA).

## 3. Results

Table 1 presents the general characteristics of study participants. Of our study population, 22% of alcohol-consuming men and 13.4% of alcohol-consuming women reported AmED consumption in the past 12 months. The mean number of negative alcohol-related consequences experienced by AmED users was 3.111 (SD: 1.560) for men and 2.678 (SD: 1.619) for women. The mean number of consequences experienced by non-AmED users was 2.421 (SD: 1.511) for men and 2.581 (SD: 1.611) for women.

Table 2 shows the association between AmED consumption and negative alcohol-related consequences after controlling for all covariates. Compared to non-AmED users, who were the reference group, AmED users experienced a greater number of negative alcohol-related consequences (men β: 0.804, *p* ≤ 0.0001; women β: 0.522, *p* ≤ 0.0001). Compared to individuals who consume alcohol less than once a month, individuals consuming alcohol in greater amounts, e.g., more than four times per week, experienced greater numbers of alcohol-related consequences (men β: 1.694, *p* ≤ 0.0001; women β: 1.560, *p* ≤ 0.0001). Compared to individuals who consume one to two drinks per drinking session, individuals consuming greater amounts of alcohol per drinking session, e.g., more than 10 drinks per drinking session, experienced greater numbers of alcohol-related consequences (men β: 1426, *p* ≤ 0.0001; women β: 1.533, *p* ≤ 0.0001).

Table 3 presents the results of the subgroup analysis examining the combined effects of drinking frequency/intake and AmED use on the number of alcohol-related consequences experienced for men and women. Compared to non-AmED users, who were the reference group, for men, individuals consuming alcohol less than once a month (β: 0.716, *p* = 0.000), once a month (β: 1.155, *p* ≤ 0.0001), two to four times per month (β: 0.806, *p* ≤ 0.0001), or two to three times per week (β: 0.606, *p* = 0.003) experienced greater numbers of alcohol-related consequences when they were users of AmED. Men who consumed alcohol once a month had the highest coefficient beta difference to non-users (β: 1.155, *p* ≤ 0.0001).

Consuming alcohol more than four times per week was not associated with negative alcohol-related consequences, according to AmED consumption. Relative to non-AmED users, regarding the number of drinks per drinking session, individuals consuming three to four drinks per drinking session (β: 1.012, *p* ≤ 0.0001), five to six drinks per drinking session (β: 0.868, *p* ≤ 0.0001), seven to nine drinks per drinking session (β: 0.517, *p* = 0.009), or more than 10 drinks per drinking session (β: 0.777, *p* ≤ 0.0001) on average experienced a greater number of alcohol-related consequences when users of AmED. Consuming one to two drinks per drinking session was not associated with negative alcohol-related consequences, according to AmED consumption. Consuming three to four drinks per drinking session resulted in the greatest coefficient beta difference to non-users (β: 1.155, *p* ≤ 0.0001).

For women, relative to non-AmED users, individuals consuming alcohol less than once a month (β: 1.019, *p* ≤ 0.0001), two to four times per month (β: 0.508, *p* = 0.003), or two to three times per week (β: 0.540 *p* = 0.038) experienced greater numbers of alcohol-related consequences when users of AmED. Consuming alcohol once a month or more than four times per week was not associated with negative alcohol-related consequences, according to AmED consumption. Women who consumed alcohol less than once a month had the highest coefficient beta difference to non-users (β: 1.019, *p* ≤ 0.0001).

Relative to non-AmED users, regarding the number of drinks per drinking session, individuals consuming one to two drinks per drinking session (β: 0.776, *p* = 0.000), three to four drinks per drinking session (β: 0.993, *p* ≤ 0.0001), five to six drinks per drinking session (β: 0.589, *p* = 0.014), or seven to nine drinks per drinking session (β: 0.715, *p* = 0.009) on average experienced a greater number of alcohol-related consequences when users of AmED. Consuming more than 10 drinks per drinking session was not associated with negative alcohol-related consequences, according to AmED consumption. Consuming three to four drinks per drinking session resulted in the greatest coefficient beta difference to non-users (β: 0.993, *p* ≤ 0.0001).

**Figure 1:**
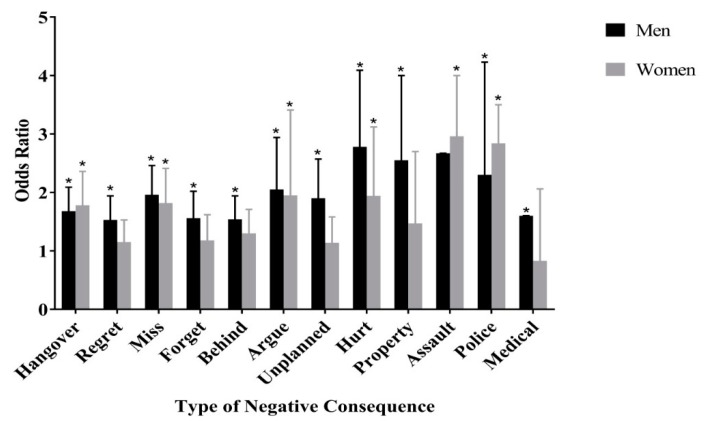
Odds of negative alcohol-related consequences according to AmED use by sex (controlled for alcohol consumption frequency, number of drinks per drinking session, year level, major, grade point average (GPA), spending allowance, smoking status, stress level, depressive thoughts, suicidal thoughts, suicidal attempt, underage drinking experience, and number of clubs/organizations).

Finally, regarding the type of consequence experienced, Figure 1 shows results of the logistic regression analysis examining the association between AmED use and type of negative alcohol-related consequence. It conveys that for men, AmED use is associated with increased odds of all twelve consequences: have a hangover (OR: 1.68, 95% CI: 1.36–2.09), do something you regret (OR: 1.53, 95% CI: 1.20–1.94), miss a class (OR: 1.96, 95% CI: 1.56–2.46), forget where you were or what you did (OR: 1.56, 95% CI: 1.20–2.02), get behind in school work (OR: 1.54, 95% CI: 1.23–1.94), argue with friends (OR: 2.05, 95% CI: 1.42–2.94), engage in unplanned sexual activity (OR: 1.90 95% CI: 1.40–2.57), get hurt or injured (OR: 2.78, 95% CI: 1.89–4.09), damage property (OR: 2.55, 95% CI: 1.11–5.83), be sexually assaulted (OR: 4.57, 95% CI: 2.35–8.88), get into trouble with campus or local police (OR: 2.30, 95% CI: 1.25–4.23), or require medical treatment (OR: 1.60, 95% CI: 1.37–1.96).

For women, AmED use was associated with increased odds of a fewer number of consequences: have a hangover (OR: 1.78, 95% CI: 1.34–2.36), miss a class (OR: 1.82, 95% CI: 1.37–2.41), argue with friends (OR: 1.95, 95% CI: 1.11–3.41), get hurt or injured (OR: 1.94, 95% CI: 1.21–3.12), be sexually assaulted (OR: 8.10, 95% CI: 2.40–27.33), or get into trouble with campus or local police (OR: 2.84, 95% CI: 1.48–5.45). However, AmED consumption was not associated with the following consequences: do something you regret, forget where you were or what you did, get behind in school work, engage in unplanned sexual activity, damage property, require medical treatment.

## 4. Discussion

The present study sought to investigate the association between alcohol mixed with energy drinks and negative alcohol-related consequences among college students. AmED consumption was found to increase the average number of negative alcohol-related consequences experienced by both men and women. Such results were in alignment with the existing body of literature that has found increased odds of negative alcohol-related consequences for AmED consumers relative to non-consumers [6,9,11,15].

For men, AmED consumption was associated with increased odds of the following alcohol-related consequences: have a hangover, do something you regret, miss a class, forget where you were or what you did, get behind in school work, argue with friends, engage in unplanned sexual activity, get hurt or injured, damage property, be sexually assaulted, or get into trouble with campus or local police. As for women, AmED was associated with increased odds of a fewer number of consequences, as follows: have a hangover, miss a class, argue with friends, get hurt or injured, be sexually assaulted, and get in trouble with campus or local police.

Such results were both consistent and inconsistent with previous studies. Studies have had mixed results regarding sex-specific consequences, especially when it comes to sexual activity. Some researchers state that AmED consumption is associated with sexual victimization for men, but not women [18], while other studies have found that AmED consumption increases odds of casual sex [19], being taken advantage of sexually [20], and overall risky sexual behavior, e.g., sex without protection [21] for both sexes.

Previous studies have also noted increased risk of medical treatment requirement among AmED users, especially high-sensation seeking males who score highly on the Brief Sensation-Seeking Scale (BSSS) (α = 0.81) [22]. This was somewhat consistent with the findings of our study, which found increased odds of medical treatment among males but not among females. Also, both male and female AmED consumers were at increased likelihood of being cautioned, restrained, charged, and/or fined by the police compared to non-consumers in both previous studies and our investigation [23].

Regarding drinking frequency and amount, there has been debate about the combined effects of AmED consumption and drinking amount on the risky behaviors of consumers. In our investigation, AmED users experienced the greatest number of alcohol-related consequences when alcohol consumption frequency was low (less than once a month/once a month) and the number of drinks per drinking session were moderate (three to four drinks per drinking session). This was an interesting finding, as while many studies have found that high-risk drinking results in more alcohol-related consequences than low-risk or moderate-risk drinking [4,9], few studies have found that low-risk or moderate-risk drinking causes more risky behavior among AmED consumers.

While there are many plausible explanations for this phenomenon, one explanation is that moderate amounts of AmED consumption can cause drinkers to believe that they are less impaired, resulting in greater risk-taking and negative alcohol-related consequences. Howland and colleagues state that social drinkers who believe caffeine will counteract impairment from alcohol actually show greater impairment [24]. However, this is not true; in a randomized clinical trial, Ligouri and Robinson found that individuals who consumed a capsule containing 200 or 400 mg of caffeine followed by alcohol (0.6 g/kg ethanol) claimed to feel “alert”, but showed no improvements in choice reaction time or body sway compared to non-AmED consumers [25]. Thus, AmED consumers who drink in low or moderate amounts may incorrectly perceive that they are capable of certain risk-taking behaviors, which results in more consequences than non-AmED consumers.

Another viable explanation is that AmED users who consume alcohol in low or moderate amounts may more often be in social settings when they drink than high-risk drinkers, where individuals are prone to participating in risky behaviors with others. For example, in a mathematical design of college students’ drinking environments and drinking amount, Mubayi and colleagues found that light and moderate drinkers are more likely to drink at social (off-campus parties, bars, etc.) than non-social settings (outdoors, residence halls, etc.), whereas heavy drinkers enjoy drinking at both social and non-social settings [26]. It is plausible that individuals who consume alcohol less than once a month or once a month do so at parties or social environments, while individuals who drink greater amounts of alcohol do so at both social and non-social settings. Energy drink industries traditionally sponsor fraternities by supplying them with products in exchange for endorsement, and students who consume AmED always mainly report social motives such as “it was being served at a party,” or “it was the only mixer available” as their reason for consuming AmED [5]. Therefore, low or moderately drinking students may be more likely than high-risk drinkers to be in an environment where they are consuming AmED and at greater risk of partaking in risky behaviors with friends or social acquaintances in proportion to high-risk drinkers. Thus, because of these factors, it is possible that the difference between AmED users and non-users is less for those who drink more often or more per occasion.

The present results of our study should be interpreted in light of a few limitations. An important limitation of our investigation is that there were no questions on frequency of AmED consumption or amount/type of AmED consumed per drinking session in our survey instrument. AmED consumption was only measured through experience in the last 12 months, which resulted in the grouping of all students with AmED consumption, regardless of frequency or amount, together. Future studies should control for these variables so that heterogeneity is accounted for.

Also, our study is cross-sectional in design; thus, caution should be exercised in interpreting causality between AmED consumption and alcohol use. Furthermore, although AmED consumption is trending in South Korea, the overall rate of college students who reported consuming AmED were relatively lower in our study population than the statistics reported in Western populations. Different cultural and social motives should be taken into consideration when interpreting this phenomenon. Likewise, all reports of negative alcohol-related consequences experienced were self-reported. For some consequences, more reliable methods of evaluating experience may have existed, e.g., police reports for misdemeanors/attendance scores for missing class, etc., however, due to the design of our study, we were unable to measure these consequences with more reliable measures. Furthermore, various biases may have emerged from our sampling and surveying methods. A small number of Christian colleges that were originally in our sample declined our request for participation because of their principles regarding abstaining from drinking and thus had to be replaced with non-Christian colleges. Because of the face-to-face method that we employed for accuracy of obtaining responses to complicated or personal questions, there may have been response biases related to social desirability. The majority of questions in our survey instrument required students to think about their drinking behaviors in the last 12 months or so, which likely resulted in recall bias. Finally, although we included numerous lifestyle covariates as potential confounders, the limited nature and number of questions in our instrument made it difficult in other confounding variables related to health, socio-demographics, gene-environment, and lifestyle to be measured and controlled, e.g., we were unable to control for drug use because it was not part of our questionnaire.

Despite these limitations, our study also has several strengths. Few studies have examined AmED consumption and its effects among a nationally representative sample of college students in South Korea or taken an epidemiological approach to see the combined effects of AmED consumption and drinking behavior on negative alcohol-related consequences according to sex. Furthermore, our results show that AmED use may have clinical utility as a screening tool for detecting risky alcohol-related behaviors that harm college students and their respective communities.

For policy-makers, such findings suggest that strategic measures are necessary to reduce AmED accessibility. Patrick and colleagues suggest regulations with bars and/or licensed establishments in the vicinity of colleges that limit AmED sales per person and/or sales of drinks combining caffeine and alcohol [12]. General campus alcohol policies that forbid the consumption of AmED on campus and educate students about the hazardous effects of co-ingesting alcohol and caffeine may also be effective [27]. Finally, improved labeling regarding the caffeine content of beverages and the harms of co-ingestion with alcohol are recommended for all over-the-counter energy drinks to reduce AmED consumption not only among college students but also among adolescents and the general population [28].

## 5. Conclusions

Our study has found that both male and female college students who consume AmED experience a greater number of negative alcohol-related consequences than those who do not. Rather than high-risk drinkers, moderate drinkers who consume alcohol infrequently and/or in low amounts may experience more consequences when consumers of AmED. More research is required to understand why low-risk or moderate-risk drinkers experience more negative alcohol-related consequences when AmED users than high-risk drinkers. Researchers, educators, and policy-makers are encouraged to further investigate and target such students when creating campus alcohol initiatives and education programs to alleviate these problems.

## Figures and Tables

**Table 1:** Sociodemographic characteristics of study participants and negative alcohol-related consequences.

	Negative Alcohol-Related Consequences									
	Men					Women				
	*N*	%	Mean	SD	*p*-Value	*N*	%	Mean	SD	*p*-Value
Total	2259	49.2	2.573	1.548		2333	50.8	2.678	1.619	
AmED Use										
Yes	496	22.0	3.111	1.560	<0.0001	312	13.4	3.304	1.534	<0.0001
No	1763	78.0	2.421	1.511		2021	86.6	2.581	1.611	
Alcohol Consumption Frequency										
Less than once a month	349	15.4	1.711	1.177	<0.0001	484	20.7	1.661	1.160	<0.0001
Once a month	370	16.4	2.116	1.395		416	17.8	2.156	1.435	
2–4 times per month	915	40.5	2.577	1.510		992	42.5	2.885	1.580	
2–3 times per week	509	22.5	3.216	1.524		384	16.5	3.805	1.447	
More than 4 times per week	116	5.1	3.767	1.429		57	2.4	3.912	1.340	
Number of Drinks per Drinking Session										
1–2 drinks	142	6.3	1.310	0.764	<0.0001	274	11.7	1.336	0.841	<0.0001
3–4 drinks	313	13.9	1.802	1.154		461	19.8	1.907	1.320	
5–6 drinks	344	15.2	2.203	1.370		371	15.9	2.429	1.456	
7–9 drinks	466	20.6	2.489	1.507		464	19.9	2.940	1.599	
More than 10 drinks	994	44.0	3.163	1.556		763	32.7	3.587	1.492	
Year Level										
1	716	31.7	2.525	1.544	<0.0001	745	31.9	2.732	1.602	<0.0001
2	714	31.6	2.601	1.546		766	32.8	2.743	1.637	
3	373	16.5	2.625	1.593		407	17.4	2.713	1.669	
≥4	456	20.2	2.561	1.525		415	17.8	2.427	1.548	
Major					<0.0001					<0.0001
Humanities and Social Sciences	968	42.9	2.651	1.584		1203	51.6	2.626	1.619	
Engineering/Natural Sciences	1107	49.0	2.491	1.511		774	33.2	2.783	1.625	
Liberal Arts	184	8.1	2.658	1.564		356	15.3	2.624	1.599	
GPA										
≥4.0	332	14.7	2.455	1.457		335	14.4	2.510	1.534	
3.5–4.0	741	32.8	2.462	1.518		889	38.1	2.531	1.606	
3.0–3.5	833	36.9	2.563	1.569	<0.0001	796	34.1	2.698	1.615	<0.0001
≤3.0	353	15.6	2.941	1.595		313	13.4	3.220	1.645	
Spending Allowance										
Q1 (Low)	836	37.0	2.272	1.440		808	34.6	2.256	1.506	
Q2	567	25.1	2.459	1.503	<0.0001	634	27.2	2.691	1.615	<0.0001
Q3	438	19.4	2.872	1.596		468	20.1	2.919	1.635	
Q4 (High)	418	18.5	3.017	1.613		423	18.1	3.196	1.614	
Smoking Status										
Current Smoker	858	38.0	2.796	1.578	<0.0001	212	9.1	3.486	1.598	<0.0001
Past Smoker	155	6.9	2.826	1.636		59	2.5	3.186	1.548	
Non-Smoker	1246	55.2	2.388	1.492		2062	88.4	2.580	1.598	
Stress Level										
High	192	8.5	2.792	1.561		315	13.5	2.800	1.620	
Normal	1629	72.1	2.598	1.539	<0.0001	1703	73.0	2.703	1.629	<0.0001
Low	438	19.4	2.384	1.560		315	13.5	2.416	1.542	
Depressive Thoughts										
Yes	208	9.2	2.697	1.513	<0.0001	322	13.8	3.121	1.626	<0.0001
No	2051	90.8	2.560	1.552		2011	86.2	2.607	1.607	
Suicidal Thoughts										
Yes	49	2.2	3.102	1.711	<0.0001	83	3.6	3.241	1.679	<0.0001
No	2210	97.8	2.561	1.543		2250	96.4	2.657	1.614	
Suicidal Attempt										
Yes	23	1.0	3.348	1.799	<0.0001	22	0.9	3.136	1.859	<0.0001
No	2236	99.0	2.565	1.544		2311	99.1	2.673	1.617	
Underage Drinking Experience										
Yes	1243	55.0	2.825	1.582	<0.0001	1031	44.2	3.090	1.654	<0.0001
No	1016	45.0	2.265	1.448		1302	55.8	2.351	1.514	
Number of sororities										
None	1158	51.3	2.483	1.511	<0.0001	1202	51.5	2.665	1.621	<0.0001
One	874	38.7	2.578	1.548		912	39.1	2.667	1.619	
Two or more	227	10.0	3.013	1.663		219	9.4	2.795	1.614	

**Table 2:** Results of the linear regression analysis analyzing the effect of alcohol mixed with energy drinks (AmED) use on negative alcohol-related consequences.

	Negative Alcohol-Related Consequences *					
	Men			Women		
	β	SD	*p*-Value	β	SD	*p*-Value
AmED Use						
Yes	0.804	0.094	<0.0001	0.522	0.106	<0.0001
No	Ref.			Ref.		
Alcohol Consumption Frequency						
Less than once a month	Ref.			Ref.		
Once a month	0.255	0.132	0.054	0.304	0.112	0.007
2–4 times per month	0.508	0.121	<0.0001	0.793	0.099	<0.0001
2–3 times per week	1.059	0.139	<0.0001	1.756	0.126	<0.0001
More than 4 times per week	1.694	0.202	<0.0001	1.560	0.244	<0.0001
Number of Drinks per Drinking Session						
1–2 drinks	Ref.			Ref.		
3–4 drinks	0.349	0.176	0.048	0.254	0.126	0.045
5–6 drinks	0.632	0.180	0.001	0.590	0.136	<0.0001
7–9 drinks	0.839	0.178	<0.0001	0.965	0.133	<0.0001
More than 10 drinks	1.426	0.172	<0.0001	1.533	0.131	<0.0001
Year Level						
1	Ref.			Ref.		
2	0.077	0.093	0.408	−0.099	0.085	0.242
3	0.063	0.118	0.597	0.071	0.106	0.501
≥4	0.038	0.115	0.744	−0.174	0.109	0.112
Major						
Humanities and Social Sciences	Ref.			Ref.		
Engineering / Natural Sciences	0.045	0.094	0.636	0.143	0.091	0.118
Liberal Arts	0.328	0.159	0.039	−0.038	0.114	0.739
GPA						
≥4.0	Ref.			Ref.		
3.5–4.0	0.120	0.097	0.217	0.164	0.089	0.066
3.0–3.5	0.255	0.109	0.020	0.267	0.101	0.008
≤3.0	0.278	0.112	0.013	0.328	0.108	0.003
Spending Allowance						
Q1 (Low)	Ref.			Ref.		
Q2	0.148	0.099	0.132	0.273	0.091	0.003
Q3	0.427	0.108	<0.0001	0.389	0.101	0.000
Q4 (High)	0.439	0.111	<0.0001	0.418	0.107	<0.0001
Smoking Status						
Current Smoker	−0.030	0.087	0.730	0.387	0.128	0.003
Past Smoker	0.065	0.151	0.669	0.028	0.219	0.899
Non-Smoker	Ref.			Ref.		
Stress Level						
High	0.507	0.159	0.001	−0.008	0.143	0.956
Normal	0.314	0.096	0.001	0.088	0.104	0.398
Low	Ref.			Ref.		
Depressive Thoughts						
Yes	−0.087	0.144	0.548	0.422	0.113	0.000
No	Ref.			Ref.		
Suicidal Thoughts						
Yes	0.944	0.289	0.001	0.437	0.207	0.035
No	Ref.			Ref.		
Suicidal Attempt						
Yes	1.114	0.394	0.005	0.441	0.371	0.234
No	Ref.			Ref.		
Underage Drinking Experience						
Yes	0.260	0.080	0.001	0.380	0.074	<0.0001
No	Ref.			Ref.		
Number of sororities						
None	Ref.			Ref.		
One	0.034	0.085	0.688	−0.012	0.078	0.880
Two or more	0.435	0.138	0.002	0.010	0.128	0.937

* Contextual effect regarding all 82 colleges in our study has been controlled for all variables.

**Table 3:** Combined effects of drinking behavior and AmED use on negative alcohol-related consequences.

	AmED User			Non-User
	Negative Alcohol-Related Consequences *			
	β	SD	*p*-Value	β
Men				
Alcohol Consumption Frequency				
Less than once a month	0.716	0.196	0.000	Ref.
Once a month	1.155	0.238	<0.0001	Ref.
2–4 times per month	0.806	0.153	<0.0001	Ref.
2–3 times per week	0.606	0.201	0.003	Ref.
More than 4 times per week	0.013	0.446	0.000	Ref.
Number of Drinks per Drinking Session				
1–2 drinks	0.200	0.206	0.331	Ref.
3–4 drinks	1.012	0.221	<0.0001	Ref.
5–6 drinks	0.868	0.220	<0.0001	Ref.
7–9 drinks	0.517	0.200	0.009	Ref.
More than 10 drinks	0.777	0.155	<0.0001	Ref.
Women				
Alcohol Consumption Frequency				
Less than once a month	1.019	0.232	<0.0001	Ref.
Once a month	0.217	0.283	0.445	Ref.
2–4 times per month	0.508	0.170	0.003	Ref.
2–3 times per week	0.540	0.262	0.038	Ref.
More than 4 times per week	0.108	0.798	0.899	Ref.
Number of Drinks per Drinking Session				
1–2 drinks	0.776	0.211	0.000	Ref.
3–4 drinks	0.993	0.238	<0.0001	Ref.
5–6 drinks	0.589	0.239	0.014	Ref.
7–9 drinks	0.715	0.273	0.009	Ref.
More than 10 drinks	0.222	0.200	0.265	Ref.

* Contextual effect regarding all 82 colleges in our study has been controlled for all variables.

## References

[1] Castle I.J.P., Yi H.Y., Hingson R.W., White A.M. (2014). State Variation in Underreporting of Alcohol Involvement on Death Certificates: Motor Vehicle Traffic Crash Fatalities as an Example. J. Stud. Alcohol Drugs.

[2] Kim K., Jang S., Jeong J. (2006). Effects of Environmental Correlates on Alcohol-related Problems among Colleges. Korean J. Health Educ. Promot..

[3] Arria A.M., Caldeira K.M., Kasperski S.J., Vincent K.B., Griffiths R.R., O’Grady K.E. (2011). Energy Drink Consumption and Increased Risk for Alcohol Dependence. Alcohol Clin. Exp. Res..

[4] Marczinski C.A., Fillmore M.T., Bardgett M.E., Howard M.A. (2011). Effects of Energy Drinks Mixed with Alcohol on Behavioral Control: Risks for College Students Consuming Trendy Cocktails. Alcohol Clin. Exp. Res..

[5] O’Brien M.C., McCoy T.P., Rhodes S.D., Wagoner A., Wolfson M. (2008). Caffeinated cocktails: Energy drink consumption, high-risk drinking, and alcohol-related consequences among college students. Acad. Emerg. Med..

[6] Berger L., Fendrich M., Fuhrmann D. (2013). Alcohol mixed with energy drinks: Are there associated negative consequences beyond hazardous drinking in college students?. Addict. Behav..

[7] Park C.L., Grant C. (2005). Determinants of positive and negative consequences of alcohol consumption in college students: Alcohol use, sex, and psychological characteristics. Addict. Behav..

[8] Peacock A., Bruno R., Martin F.H. (2012). The subjective physiological, psychological, and behavioral risk-taking consequences of alcohol and energy drink co-ingestion. Alcohol Clin. Exp. Res..

[9] Johnson S.J., Alford C., Stewart K., Verster J.C. (2018). Are energy drinks unique mixers in terms of their effects on alcohol consumption and negative alcohol-related consequences?. Int. J. Gen. Med..

[10] Olive M. (2002). Interactions between taurine and ethanol in the central nervous system. Amino Acids.

[11] Patrick M.E., Evans-Polce R.J., Maggs J.L. (2014). Use of alcohol mixed with energy drinks as a predictor of alcohol-related consequences two years later. J. Stud. Alcohol Drugs.

[12] Magnezi R., Bergman L.C., Grinvald-Fogel H., Cohen H.A. (2015). A survey of energy drink and alcohol mixed with energy drink consumption. Isr. J. Health Policy.

[13] Marczinski C.A., Fillmore M.T., Henges A.L., Ramsey M.A., Young C.R. (2013). Mixing an Energy Drink with an Alcoholic Beverage Increases Motivation for More Alcohol in College Students. Alcohol. Clin. Exp. Res..

[14] Marczinski C.A., Fillmore M.T., Stamates A.L., Maloney S.F. (2016). Desire to Drink Alcohol is Enhanced with High Caffeine Energy Drink Mixers. Alcohol. Clin. Exp. Res..

[15] Wechsler H., Davenport A., Dowdall G., Moeykens B., Castillo S. (1994). Health and Behavioral Consequences of Binge Drinking in College: A National Survey of Students at 140 Campuses. JAMA.

[16] Oh S.S., Ju Y.J., Lee S., Park E.-C. (2019). Primary reason for drinking among current, former, and never flushing college students. Int. J. Environ. Res. Public Health.

[17] Park C.L. (2004). Positive and negative consequences of alcohol consumption in college students. Addict. Behav..

[18] Snipes D.J., Green B.A., Javier S.J., Perrin P.B., Benotsch E.G. (2014). The use of alcohol mixed with energy drinks and experiences of sexual victimization among male and female college students. Addict. Behav..

[19] Miller K.E. (2012). Alcohol mixed with energy drink use and sexual risk-taking: Casual, intoxicated, and unprotected sex. J. Caffeine Res..

[20] Cobb C.O., Nasim A., Jentink K., Blank M.D. (2015). The role of caffeine in the alcohol consumption behaviors of college students. Subst. Abuse.

[21] Miller K.E. (2008). Energy drinks, race, and problem behaviors among college students. J. Adolesc. Health.

[22] O’Brien M.C., McCoy T.P., Egan K.L., Goldin S., Rhodes S.D., Wolfson M. (2013). Caffeinated alcohol, sensation seeking, and injury risk. J. Caffeine Res..

[23] Peacock A., Droste N., Pennay A., Lubman D.I., Miller P., Newcombe D., Bruno R. (2015). Self-Reported Risk-Taking Behavior During Matched-Frequency Sessions of Alcohol Versus Combined Alcohol and Energy Drinks Consumption: Does Co-Ingestion Increase Risk-Taking?. Alcohol. Clin. Exp. Res..

[24] Howland J., Rohsenow D.J. (2013). Risks of energy drinks mixed with alcohol. JAMA.

[25] Liguori A., Robinson J.H. (2001). Caffeine antagonism of alcohol-induced driving impairment. Drug Alcohol Depend..

[26] Mubayi A., Greenwood P., Wang X., Castillo-Chávez C., Gorman D.M., Gruenewald P., Saltz R.F. (2011). Types of drinkers and drinking settings: An application of a mathematical model. Addiction.

[27] Reissig C.J., Strain E.C., Griffiths R.R. (2009). Caffeinated energy drinks—A growing problem. Drug Alcohol Depend..

[28] Larimer M.E., Cronce J.M. (2007). Identification, prevention, and treatment revisited: Individual-focused college drinking prevention strategies 1999–2006. Addict. Behav..

